# Nondestructive monitoring of annealing and chemical–mechanical planarization behavior using ellipsometry and deep learning

**DOI:** 10.1038/s41378-023-00529-9

**Published:** 2023-04-28

**Authors:** Qimeng Sun, Dekun Yang, Tianjian Liu, Jianhong Liu, Shizhao Wang, Sizhou Hu, Sheng Liu, Yi Song

**Affiliations:** 1 The Institute of Technological Sciences, Wuhan University, Wuhan, China; 2 School of Mechanical Science and Engineering, Huazhong University of Science and Technology, Wuhan, China; 3 Hongyi Honor College of Wuhan University, Wuhan, China; 4 School of Power and Mechanical Engineering, Wuhan University, Wuhan, China; 5 The School of Microelectronics, Wuhan University, Wuhan, China

**Keywords:** Micro-optics, Electrical and electronic engineering, Electronic devices, Computational nanotechnology

## Abstract

The Cu-filling process in through-silicon via (TSV-Cu) is a key technology for chip stacking and three-dimensional vertical packaging. During this process, defects resulting from chemical–mechanical planarization (CMP) and annealing severely affect the reliability of the chips. Traditional methods of defect characterization are destructive and cumbersome. In this study, a new defect inspection method was developed using Mueller matrix spectroscopic ellipsometry. TSV-Cu with a 3-μm-diameter and 8-μm-deep Cu filling showed three typical types of characteristics: overdishing (defect-OD), protrusion (defect-P), and defect-free. The process dimension for each defect was 13 nm. First, the three typical defects caused by CMP and annealing were investigated. With single-channel deep learning and a Mueller matrix element (MME), the TSV-Cu defect types could be distinguished with an accuracy rate of 99.94%. Next, seven effective MMEs were used as independent channels in the artificial neural network to quantify the height variation in the Cu filling in the z-direction. The accuracy rate was 98.92% after training, and the recognition accuracy reached 1 nm. The proposed approach rapidly and nondestructively evaluates the annealing bonding performance of CMP processes, which can improve the reliability of high-density integration.

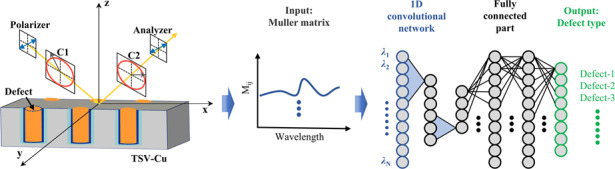

## Introduction

In recent years, three-dimensional (3D) integrated circuit (IC) technology with through-silicon via (TSV) has attracted significant attention because of its versatility, small size, and high performance. 3D IC is a technology that reduces the overall wire length and delay by vertically stacking multiple chips through high-density chip-to-chip interconnects^1–7^. TSV technology involves several processes, including etching holes in Si chips, depositing insulating/blocking/seeding layers, filling blind holes with Cu conductors, removing the backside Si and Cu overlay films via chemical–mechanical planarization (CMP) to expose Cu microcartridges, and ball bonding^8,9^. Several researchers have investigated the TSV manufacturing process. The microcharacterization and nanocharacterization of various processes have also received considerable attention in improving the reliability of 3D ICs.

Significant issues in TSV technology include residual stress, extrusion, cracking, delamination, and Cu leaks, leading to severe reliability problems, such as warping of chips or wafers, interface delamination, cracking, and weak internal wire contact. These problems mainly result from the significant difference in the coefficient of thermal expansion between the surrounding silicon substrate and the TSV filler metal^10–12^. Recently, studies on the Cu-filling process in TSV (TSV-Cu) protrusion have been conducted^13–15^. In these processes, detailed physical analyses are required because of the considerable impact of TSV-Cu protrusion on the reliability of the final 3D stacking. Many studies have been conducted to investigate annealing Cu protrusion and CMP dishing^16–23^. Cu protrusion and excessive dishing reduce the reliability of 3D stacking. In TSV manufacturing, it is crucial to control the CMP speed, annealing time, and temperature. Insufficient or excessive wafer polishing can lead to leaks and shorts, making the chips defective. Residual stress, interface delamination, and cracking occur when the annealing time or temperature is insufficient or excessive^6,24–26^. However, real-time nondestructive characterization methods for Cu protrusions and dishing remain limited.

Nondestructive characterization of TSV-Cu in a production line has not been extensively investigated. When many defects appear during the TSV process, the detection time obtained using conventional methods is unsatisfactory^5,9^. The period and critical size of the TSV-Cu structure prevail on the microscale for both vertical and horizontal dimensions. However, during CMP and annealing processes, the accuracy of the z-direction of Cu must be controlled at the nanoscale^5,15,24^. Cross-scale defect characterization in the z-direction of TSV-Cu structures is therefore a significant challenge.

In this study, an ellipsometry measurement method was developed for the characterization of TSV-Cu. The rigorous coupled-wave analysis (RCWA) algorithm was employed to calculate the reflection electric field of TSV-Cu with different dimensions of Cu filling in the z-direction at different wavelengths. The corresponding Mueller matrices were calculated by modulating the incident and reflective electric fields, as described in the “Methods” section. Deep learning with Mueller matrix datasets (see the “Methods” section for details) was used to distinguish the different TSV-Cu defects. The effects of the annealing temperature and time on the Cu grain size were experimentally studied, and the defect size of TSV-Cu was quantified using a multichannel deep-learning method. A single Mueller matrix element (MME) was replaced with multiple MMEs as a dataset to improve the stability and accuracy of the quantitative TSV-Cu size.

## Results and discussion

### Defect classification of TSV-Cu

**Fig. 1:**
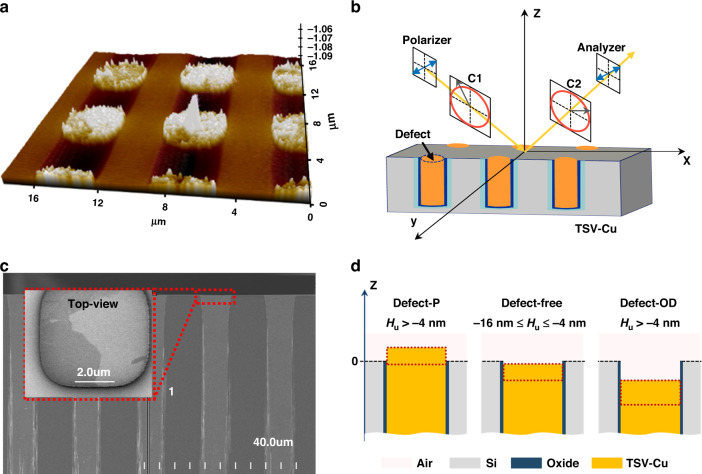
Images of TSV-Cu and schematics of defect classification. **a** Atomic force microscopy image of 3-μm-diameter and 8-μm-deep TSV-Cu structure. **b** Optical simulation model of the TSV-Cu structure. **c** Cross-section of TSV-Cu and top-view image (insert) of Cu filling. **d** Schematic of defect division

Figure 1a shows an atomic force microscopy image of a 3-μm-diameter and 8-μm-deep TSV-Cu structure after annealing at 250 °C for 8.5 h. An optical model was established based on this structure, as shown in Fig. 1b. The model comprised 20 nm Ta as a blocking layer, 200 nm SiO_2_ as an insulating layer, a silicon substrate, and a Cu-filling layer with a diameter of 3 μm and depth of 8 μm. The Cu-filling period was 6 μm, the incidence angle was fixed at 45°^27^, and the wavelength range was 400–1000 nm. The scanning electron microscopy images of the TSV-Cu cross-section are shown in Fig. 1c. The red dotted line represents the areas where different types of defects appeared in this study. The top view of the Cu-filled TSV is shown by the red dotted line (Fig. 1c). During the annealing process, when the temperature is lower than 200 °C, the height of the upper surface of the Cu-filling (***H***_**u**_) changes due to the significant mismatch of the coefficient of thermal expansion (CTE) between the Cu-filling and silicon substrate. Due to the higher CTE of Cu, the expansion of Cu is greater than that of silicon. At this temperature, there is almost no change in the microstructure of Cu-filling. The recrystallization of Cu-filling occurs at approximately 200–250 °C, at which point the Cu grain is refined. Once the annealing temperature exceeds 250 °C, the grain grows significantly as driven by the total free energy^3^. ***H***_**u**_ is positively correlated with the grain size^14^. Therefore, ***H***_**u**_ increases subsequentially. During the CMP process, the removal of the barrier layer and insulating layer is slower than that of Cu filling. Through this process, ***H***_**u**_ decreases subsequentially. When ***H***_**u**_ is excessively large or small, there is a risk of cracks and voids, respectively. Under the effect of poor bonding quality, the electric and thermal resistance of TSV-Cu increase, and the surface bonding strength decreases. Therefore, a reasonable ***H***_**u**_ process window (−16 to −4 nm) is proposed in this work to achieve void-free and reliable interconnection^7,13^. A schematic of the defect types is shown in Fig. 1d. The upper surface of Si was regarded as the reference plane of 0 nm. When ***H***_**u**_ exceeded 0 nm or was lower than 4 nm in the z-direction, it was considered a protrusion defect (defect-P). ***H***_**u**_ ranging between 4 and 16 nm was considered free from defects (defect-free). ***H***_**u**_ exceeding 16 nm indicated an overdishing defect (defect-OD). Defect-P and defect-OD caused extrusion cracking and incomplete contact during annealing bonding, respectively. The actual process window (4–16 nm) was expected to have a higher upper limit because no increase in the dielectric bond strength was assumed.

**Fig. 2:**
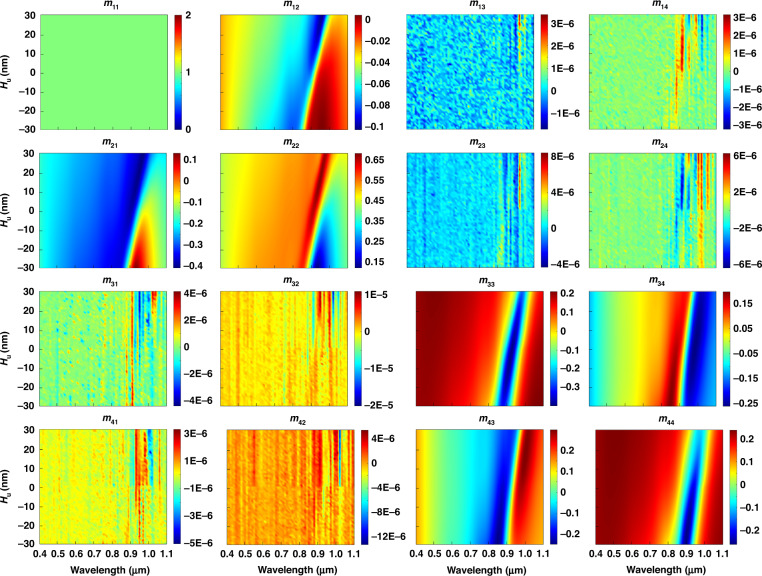
Fifteen MMEs at different wavelengths after simulation and normalization of *m*_11_. The ordinate of each photograph represents the size of the Cu filling in the z-direction (unit: nm), and the abscissa represents the wavelength (unit: μm)

Figure 2 shows the 4 × 4 MMEs at different wavelengths after ellipsometry and normalization of *m*_11_. The ordinate of each photograph represents ***H***_**u**_, and the abscissa represents the wavelength. The off-diagonal MMEs were nearly zero because the structure of TSV-Cu was isotropic^28^. The sensitivity of the MME, *m*_12_, with a change in the structure, increased at 0.7–1.0 μm wavelengths. The sensitivities of *m*_21_ and *m*_22_ with structural change increased at 0.85–1.05 μm wavelengths. The sensitivities of *m*_33_ and *m*_44_ with structural change increased at wavelengths of 0.80–1.05 μm. In addition, the sensitivities of *m*_34_ and *m*_43_ with structural change increased at 0.80–0.95 μm wavelengths. We selected seven effective MMEs (*m*_12_, *m*_21_, *m*_22_, *m*_33_, *m*_34_, *m*_43_, and *m*_44_) as the sample set for defect classification, ignoring the off-diagonal MMEs. The effective MMEs corresponding to 13 structures with ***H***_**u**_ ranging from −4 to −16 nm (intervals of 1 nm), −17 to −29 nm (intervals of 1 nm), and −3 to 9 nm (intervals of 1 nm) and the 300 noise dataset of each structure were adopted as the artificial neural network (ANN) datasets representing the defect-free, defect-OD, and defect-P topologies, respectively.

**Fig. 3:**
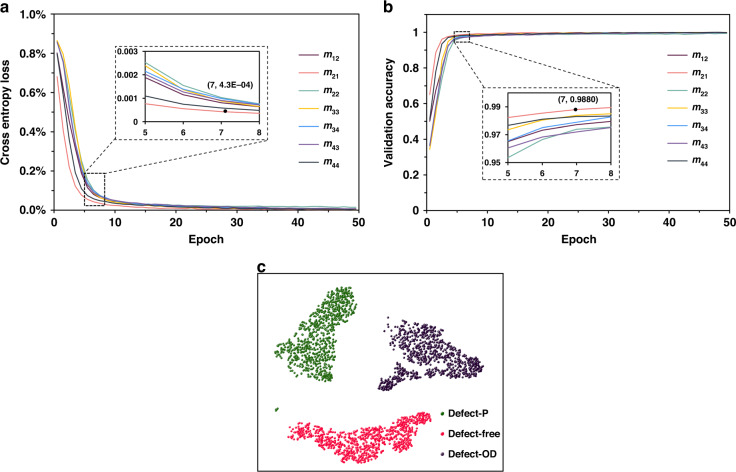
Training quality and defect classification accuracy of the ANN trained on effective Mueller matrix spectra. **a** Cross-entropy loss of training for TSV-Cu defects using *m*_12_, *m*_21_, *m*_22_, m_33_, *m*_34_, m_43_, and *m*_44_. **b** Verification accuracy of training for TSV-Cu defects using *m*_12_, *m*_21_, *m*_22_, *m*_33_, *m*_34_, *m*_43_, and *m*_44_. **c** t-SNE plot of *m*_12_ training sets for defect classification

Figures 3a and 3b show the cross-entropy loss and verification accuracy of different MMEs in the defect classification trained network, respectively. The training convergence speed of *m*_12_ was faster than those of the other elements, with an increase in training time. The Mueller matrices can be decomposed using the Lu–Shipman polar decomposition method^29^:

1 $$D = \frac{{\sqrt {{\it{m}}_{12}^2 + {\it{m}}_{13}^2 + {\it{m}}_{14}^2} }}{{{\it{m}}_{11}}}$$

where *D* is the dichroic scalar of the Mueller matrices after polar decomposition, and $D \approx {\it{m}}_{12}$. Therefore, the diattenuation of TSV-Cu with different ***H***_**u**_ values showed obvious changes. In defect classification training using *m*_12_, the verification accuracy at the seventh epoch was 99.80%, and the corresponding cross-entropy loss was 0.00043. Hence, only a single MME was required as a sample set to complete the defect classification using deep learning. t-distributed stochastic neighbor embedding (t-SNE) was employed to analyze the defect types, as shown in Fig. 3c^30^. Different scatter colors represent different defect types, and each point represents the simulation data with 10% noise randomly generated by *m*_12_ (Fig. 2). The separation of the three clusters was more evident (Fig. 3c), indicating that the defect types could be distinguished precisely. *m*_12_, *m*_21_, *m*_22_, *m*_33_, *m*_34_, *m*_43_, and *m*_44_ achieved defect classification accuracy values of 99.53%, 99.75%, 98.69%, 99.80%, 99.94%, 99.77%, and 99.66%, respectively.

### Quantification of TSV-Cu defect size using multichannel deep learning

**Fig. 4:**
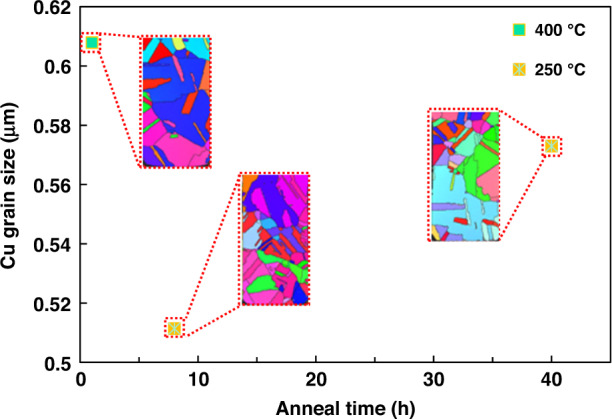
TSV-Cu average grain sizes at different annealing temperatures and times

During the TSV process, annealing was applied to make the Cu grains more homogeneous and to release the residual stress. The Cu grain sizes were measured using electron backscatter diffraction at different annealing temperatures and times to show the need for defect quantification and required quantization accuracy (Fig. 4). Higher temperatures and longer times facilitate grain growth, produce Cu protrusions, and release residual stress. Minute grains appeared when the temperature and time were 250 °C and 40 h, respectively. Two or more nuclei were formed during the extended annealing times. Therefore, the fusing and growth times of the grains are insufficient when the annealing time is extremely short. When the annealing time was very long, small grains were formed owing to repeated nuclei precipitation, leading to inadequate annealing. When annealed at 250 °C, the average Cu grain size increased by 60 nm from 9 to 40 h. Therefore, it is necessary to adjust the annealing time and temperature to control ***H***_**u**_ during annealing bonding. The proposed metrology method should be able to distinguish 10% of the process window to effectively monitor the TSV-Cu process. In this case, the recognition accuracy in the z-direction should exceed 1.2 nm to more accurately characterize ***H***_**u**_ in TSV-Cu and be defect-free within the 12 nm process window. In this section, we attempted to increase the recognition accuracy to 1 nm.

**Fig. 5:**
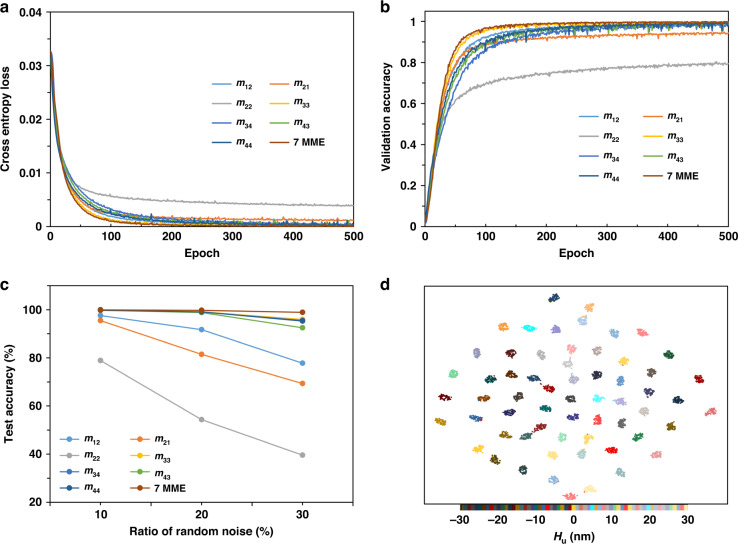
Training quality and defect classification accuracy of different MMEs. **a** Cross-entropy loss of single-channel and multichannel with 10% random noise. **b** Validation accuracy of single-channel multichannel with 10% random noise. **c** Test accuracy of single-channel and multichannel under 10%, 20%, and 30% random noises. **d** t-SNE plot of seven MME training sets for 61 ***H***_**u**_

**Fig. 6:**
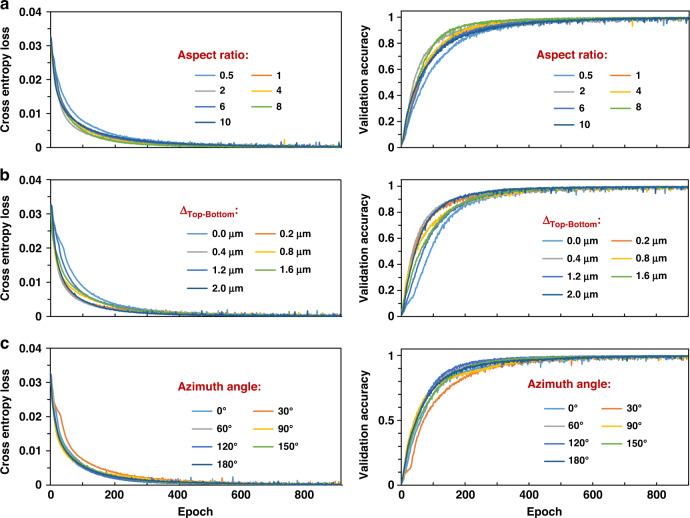
Training quality of different morphologies and different measurement conditions. Cross-entropy loss and validation accuracy using 7 MMEs of **a** different aspect ratios, **b** different Δ_Top-Bottom_, and **c** different azimuth angles

The training and testing accuracy and stability of deep learning must be improved to achieve a distinguishing accuracy level of 1 nm. In this study, a multichannel deep learning method was applied, in which seven effective MMEs were utilized. Because the actual characterization system causes random errors, random noise was added to the MMEs of each structure. Figure 5a shows the cross-entropy loss of a single channel (one MME) and multiple channels (seven MMEs) under 10% random noise, and Fig. 5b depicts the validation accuracy. The training object consisted of 61 labels, including ***H***_**u**_ ranging from –30 to 30 (in intervals of 1 nm). The convergence rate of the multichannel training cross-entropy loss and validation accuracy was faster than that of single-channel training. Figure 5c depicts the test accuracy of the single-channel and multichannel methods with different random noises. The test accuracy of the single channel was significantly influenced by the increase in noise, whereas its influence on the multichannel was less. When the random noise increased from 10% to 30%, the test accuracy of the multichannel changed by only 1.01%. When the random noise increased to 30%, the multichannel deep-learning test accuracy reached 98.92%. t-SNE was employed to quantify the size to intuitively evaluate the difference in the TSV-Cu size (Fig. 5d). In the t-SNE scatter plot, clusters with good separation indicate that the TSV-Cu size can be distinguished clearly, whereas the adjacent and overlapping clusters indicate very similar datasets. The effects of different morphologies and different measurement conditions on the measurement sensitivity were discussed using seven MMEs (Fig. 6). One such effect is the aspect ratio of TSV-Cu. The diameter of the Cu-filling is 3 μm, and the aspect ratios are 0.5, 1, 2, 4, 6, 8, and 10 (Fig. 6a). Another such effect is the critical dimension difference between the top and bottom (Δ_Top-Bottom_) of the Cu-filling. The diameter of the Cu-filling is 3 μm, the aspect ratio is 1, and the Δ_Top-Bottom_ values are 2 μm, 1.6 μm, 1.2 μm, 0.8 μm, 0.4 μm, 0.2 μm, and 0 μm (Fig. 6b). The third is that the azimuth angles of incident light include 0°, 30°, 60°, 90°, 120°, 150°, and 180° (Fig. 6c). As seen from Table 1, with the change in the TSV morphologies and measurement conditions, the test accuracies fluctuate by no more than 1%. In addition, all the cross-entropy losses and validation accuracies converge to 0 and 1, respectively, as shown in Fig. 6. Hence, multichannel deep learning using seven MMEs can quantify the ***H***_**u**_ of TSV-Cu with a quantization accuracy of 1 nm and is not affected by random errors, aspect ratios, Δ_Top-Bottom_ or azimuths.

**Table 1:** The test accuracies with different morphologies and different measurement conditions

Aspect ratio	0.5	1	2	4	6	8	10
Test accuracy (%)	99.40	99.96	99.62	99.89	99.69	99.32	99.98
Δ_Top-Bottom_ (μm)	0	0.2	0.4	0.8	1.2	1.6	2
Test accuracy (%)	99.78	99.82	98.87	99.65	99.98	99.94	99.96
Azimuth angle (°)	0	30	60	90	120	150	180
Test accuracy (%)	99.75	99.58	99.98	99.96	99.96	99.93	99.54

*MPL* max-pooling layer

## Conclusion

The polarization of light is highly sensitive to TSV-Cu particles of different sizes. In this study, three defect process windows of TSV were selected, and the Mueller matrix of TSV-Cu was calculated using Fourier series and ellipsometry. The effective MME and single-channel deep learning distinguished the defect types of TSV-Cu with an accuracy of more than 99%. Multichannel deep learning based on seven MMEs quantified the ***H***_**u**_ of TSV-Cu and achieved a recognition accuracy level of 1 nm. Random noise significantly impacts quantization accuracy, reaching 98.92% when random noise increases to 30%. Multichannel deep learning is more accurate and stable and less affected by random noise, aspect ratios, Δ_Top-Bottom_, and azimuths, which can improve the reliability of high-density integration in micro- and nanofabrication technologies.

## Methods

### Optical simulation and calculation of the Mueller matrix

The modulation method of the incident optical wave can be considered natural light transmitting a stationary linear polarizer and a quarter-wave plate in turn, and the fast-axis rotation angle is *θ*. The Stokes vector of natural light is $$\scriptstyle S_0 = \left[ {\begin{array}{*{20}{c}} 1 \\ {\begin{array}{*{20}{c}} 0 \\ 0 \\ 0 \end{array}} \end{array}} \right]$$. The Mueller matrix of the linear polarizer is $$\scriptstyle M_{{{\mathrm{P}}}} = \frac{1}{2}\left[ {\begin{array}{*{20}{c}} {\begin{array}{*{20}{c}} 1 & 1 \\ 1 & 1 \end{array}} & {\begin{array}{*{20}{c}} 0 & 0 \\ 0 & 0 \end{array}} \\ {\begin{array}{*{20}{c}} 0 & 0 \\ 0 & 0 \end{array}} & {\begin{array}{*{20}{c}} 0 & 0 \\ 0 & 0 \end{array}} \end{array}} \right]$$ and that of the quarter-wave plate is $$\scriptstyle M_{{{{\mathrm{C1}}}}} = \left[ {\begin{array}{*{20}{c}} {\begin{array}{*{20}{c}} 1 & 0 \\ 0 & 1 \end{array}} & {\begin{array}{*{20}{c}} 0 & 0 \\ 0 & 0 \end{array}} \\ {\begin{array}{*{20}{c}} 0 & 0 \\ 0 & 0 \end{array}} & {\begin{array}{*{20}{c}} 0 & 1 \\ { - 1} & 0 \end{array}} \end{array}} \right]$$. The rotation matrix is $$\scriptstyle R(\theta ) = \;\left[ {\begin{array}{*{20}{c}} {\begin{array}{*{20}{c}} 1 & 0 \\ 0 & {\cos 2\theta } \end{array}} & {\begin{array}{*{20}{c}} 0 & 0 \\ {\sin 2\theta } & 0 \end{array}} \\ {\begin{array}{*{20}{c}} 0 & { - \sin 2\theta } \\ 0 & 0 \end{array}} & {\begin{array}{*{20}{c}} {\cos 2\theta } & 0 \\ 0 & 1 \end{array}} \end{array}} \right]$$, where *θ* is the fast-axis rotation angle of the first quarter-wave plate with values of 0°, 5°, 10°, 15°,…, 170°, 175°, and 180°. Therefore, the Stokes vector of incident light on the sample, *S*_in_, can be defined as follows.

2 $$S_{{{{\mathrm{in}}}}} = R(5\theta ){\it{M}}_{{{{\mathrm{C1}}}}}R( - 5\theta )M_{{{\mathrm{P}}}}S_{{{\mathrm{0}}}}$$

In addition, the polarization angle and phase difference of the incident optical wave can be expressed as follows:

3 $$\Psi = - {{{\mathrm{Sign}}}}\left\{ {\sin 2\theta } \right\}\;\arcsin \sqrt {\frac{{1 - \cos ^22\theta }}{2}}$$

4 $$\Phi \; = \frac{{\arccos [\cos 2(\theta + 90^\circ )]}}{{\sqrt {1 + \cos ^22(\theta + 90^\circ )} }}$$

where Sign{*x*} = 1 if *x* > 1, Sign{*x*} = 0 if *x* = 0, and Sign{*x*} < 0 if *x* < 0. Ψ is the polarization angle of the incident optical wave, where Ψ = 0° is p-polarized and Ψ = 90° is s-polarized. Φ is the phase difference between the incident optical waves, and *θ* is the fast-axis rotation angle of the first quarter-wave plate.

The reflected electric fields, *E*_*x*_ and *E*_*y*_, and the corresponding phases of incident light after passing through TSV-Cu were calculated using the RCWA algorithm^31^. The RCWA algorithm was integrated into RSoft Diffractmod (RSoft Design Group, Inc., USA). The relationship between the reflected field and wavelength of the structural, optical model representing the periodic grating was calculated. $$\scriptstyle M_{{{{\mathrm{TSV - Cu}}}}} = \left[ {\begin{array}{*{20}{c}} {\begin{array}{*{20}{c}} {m_{11}} & {m_{12}} \\ {m_{21}} & {m_{22}} \end{array}} & {\begin{array}{*{20}{c}} {m_{13}} & {m_{14}} \\ {m_{23}} & {m_{24}} \end{array}} \\ {\begin{array}{*{20}{c}} {m_{31}} & {m_{32}} \\ {m_{41}} & {m_{42}} \end{array}} & {\begin{array}{*{20}{c}} {m_{33}} & {m_{34}} \\ {m_{43}} & {m_{44}} \end{array}} \end{array}} \right]$$ is the Mueller matrix of TSV-Cu. The Stokes vector reflected by the sample, *S*_*s*_, can be expressed as follows:

5 $$S_{{{\mathrm{S}}}} = \left[ {\begin{array}{*{20}{c}} {S_{{{{\mathrm{s1}}}}}} \\ {\begin{array}{*{20}{c}} {S_{{{{\mathrm{s2}}}}}} \\ {S_{{{{\mathrm{s3}}}}}} \\ {S_{{{{\mathrm{s4}}}}}} \end{array}} \end{array}} \right] = \left[ {\begin{array}{*{20}{c}} {E_{{{\mathrm{x}}}}^2 + E_{{{\mathrm{y}}}}^2} \\ {\begin{array}{*{20}{c}} {E_{{{\mathrm{x}}}}^2 - E_{{{\mathrm{y}}}}^2} \\ {2E_{{{\mathrm{x}}}}E_{{{\mathrm{y}}}}\cos \delta } \\ {2E_{{{\mathrm{x}}}}E_{{{\mathrm{y}}}}\sin \delta } \end{array}} \end{array}} \right] = M_{{{{\mathrm{TSV}}}} - {{{\mathrm{Cu}}}}}R(5\theta )M_{{{{\mathrm{C1}}}}}R( - 5\theta )M_{{{\mathrm{P}}}}S_{{{\mathrm{0}}}}$$

where *δ* is the phase difference between *E*_x_ and *E*_y_. The light reflected by the sample is remodulated as *S*_out_:

6 $$S_{{{{\mathrm{out}}}}} = \left[ {\begin{array}{*{20}{c}} {S_{{{{\mathrm{out1}}}}}} \\ {\begin{array}{*{20}{c}} {S_{{{{\mathrm{out2}}}}}} \\ {S_{{{{\mathrm{out3}}}}}} \\ {S_{{{{\mathrm{out4}}}}}} \end{array}} \end{array}} \right] = M_{{{\mathrm{A}}}}R(3\theta )M_{{{{\mathrm{C2}}}}}R( - 3\theta )S_{{{\mathrm{S}}}}$$

where *M*_*A*_ is the Mueller matrix of the analyzer, $M_{{{\mathrm{A}}}} = M_{{{\mathrm{P}}}}$. $M_{{{{\mathrm{C2}}}}}$ is the Mueller matrix of the second quarter-wave plate, $M_{{{{\mathrm{C2}}}}} = M_{{{{\mathrm{C1}}}}}\,\cdot\,{S}_{{\mathrm{out}}1}$. is the total light intensity detected by the detector. In this study, ${S}_{{\rm{out}}1}$ was expanded using the Fourier series. Because the rotation angle ratio of the incident and outgoing quarter-wave plates was 5:3, the highest order of the Fourier series was 32. Sixteen MMEs of TSV-Cu were calculated, and all elements were normalized to *m*_11_^32^.

### Deep learning

**Fig. 7:**
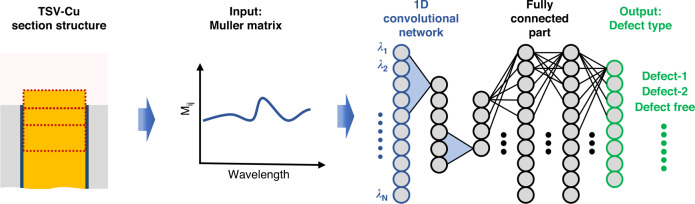
Schematic of a one-dimensional convolutional ANN

**Fig. 8:**
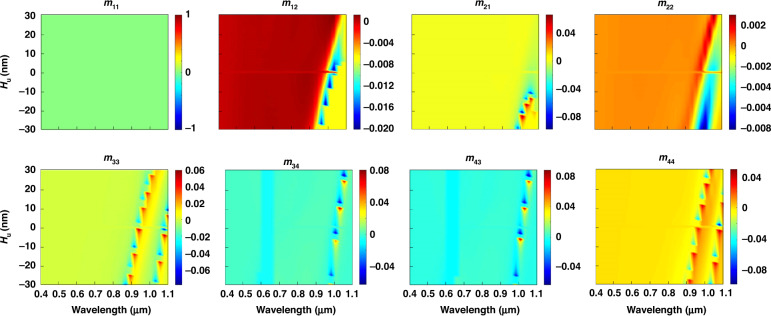
The effective spectral change rates with a surface displacement fluctuation of 0.1 nm. The ordinate of each photograph represents the size of the Cu filling in the z-direction (unit: nm), the abscissa represents the wavelength (unit: μm), and the color bar represents the spectral change rates

In this study, we developed a machine learning approach to classify defects. The convolutional neural network (CNN) was employed, matching a fully connected network (FCN) (Fig. 7). The “leaky ReLU” activation function, a preferred method for solving pattern recognition problems, was used, and it consisted of three layers^33,34^. Each CNN layer was connected to a max-pooling layer with batch normalization. The number of filters and kernel size of each channel in each CNN layer are listed in Table 2. The FCN consisted of two layers with 64 and 32 neurons and utilized “tanh” activations. Mueller matrices of different ***H***_**u**_ at different wavelengths were introduced into the ANN input layer as parallel channels, and the kernel size of each channel was seven. Each defect type was assigned to a neuron in the ANN output layer. During the training of the ANN, a Mueller matrix was introduced into the ANN input layer, and different defect types were introduced into the output layer. Hundreds or tens of sample sets for each group of structures were not produced because of the cost and time in the actual TSV-Cu production process. During the process of sample measurement, there are two random errors, including the sample manufacturing error and measurement error^35,36^. The manufacturing error of surface displacement fluctuation of 0.1 nm is inevitable in the TSV-Cu process^23^. It is difficult to obtain specific measurement error because the measurement error depends on the instrument and environment. In practice, the spectral change caused by measurement error is usually smaller than that caused by manufacturing error. The spectrum of TSV-Cu surface fluctuation (***H***_**u**_ ± 0.1 nm) was analyzed, and all effective spectral change rates were less than 10% (Fig. 8). It can be seen from Fig. 8 that only a small number of spectral change rates are close to 10%. Therefore, 60 structures were simulated, each with a Cu pillar size interval of 1 nm in the z-direction, and 300 sets of 10% noise from the Mueller matrix of each structure at different wavelengths were generated as the training dataset. Seventy percent of the Mueller matrices were used for training. The remaining 30% were used for testing. In the readout scheme, the Mueller matrix was fed into the ANN after training and propagated forward through the network to retrieve the defect types distinguished by the z-direction structure. Finally, the cross-entropy loss and validation accuracy were determined to verify the training quality of the ANN.

**Table 2:** Number of filters and kernel size of each channel in each CNN layer

Layer	Number of filters in each channel	Kernel size	Kernel size of MPL
First	32	7	2
Second	32	5	2
Third	1	3	2
